# Supplementary figures and images for: Evaluating cancer cell line and patient‐derived xenograft recapitulation of tumor and non‐diseased tissue gene expression profiles in silico
Source: Cancer Rep (Hoboken). 2023 Aug 2;6(9):e1874. doi: 10.1002/cnr2.1874 (PMC10480419; doi:10.1002/cnr2.1874)

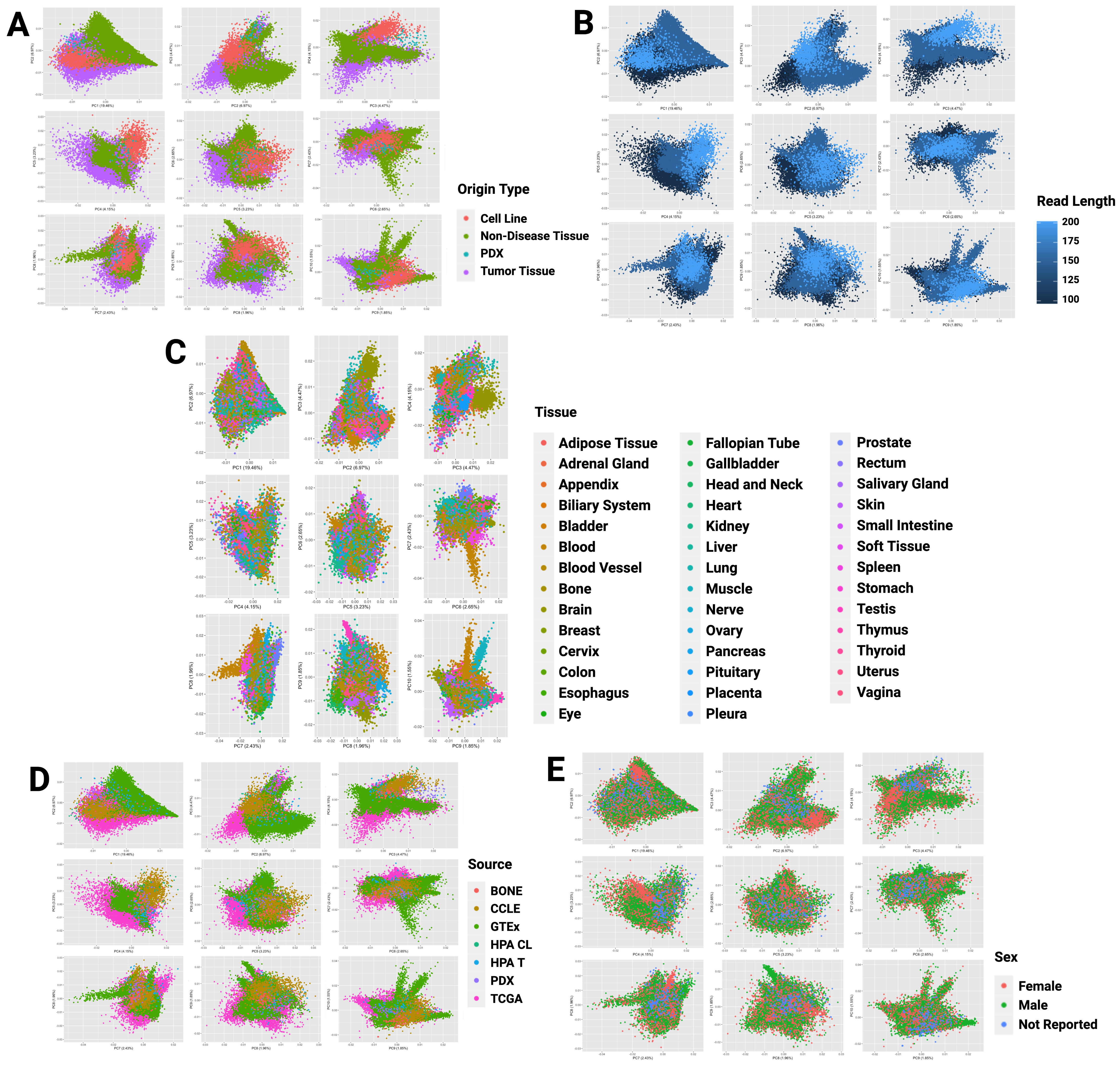

Supplement: Supplementary file 1 — FIGURE S1. A. Principal component analysis scatterplot with density rug plot of gene expression profiles from cancer cell lines (red), GBM PDX models (teal), non‐diseased tissue (green), and tumor tissue samples (purple) in principal components 1–10. B. Principal component analysis scatterplot with density rug plot of gene expression profiles from the same groups colored by read length. C. Principal component analysis scatterplot with density rug plot of gene expression profiles from the same groups colored by tissue type, regardless of disease state. D. Principal component analysis scatterplot with density rug plot of gene expression profiles from the same groups colored by the data resource samples originated from. E. Principal component analysis scatterplot with density rug plot of gene expression profiles of the same groups colored by sex of the individual that samples were originally derived from. [file CNR2-6-e1874-s009.png]

Median Correlation of Brain Model to Non-Disease  
Tissues in Each Tissue's Specific Genes

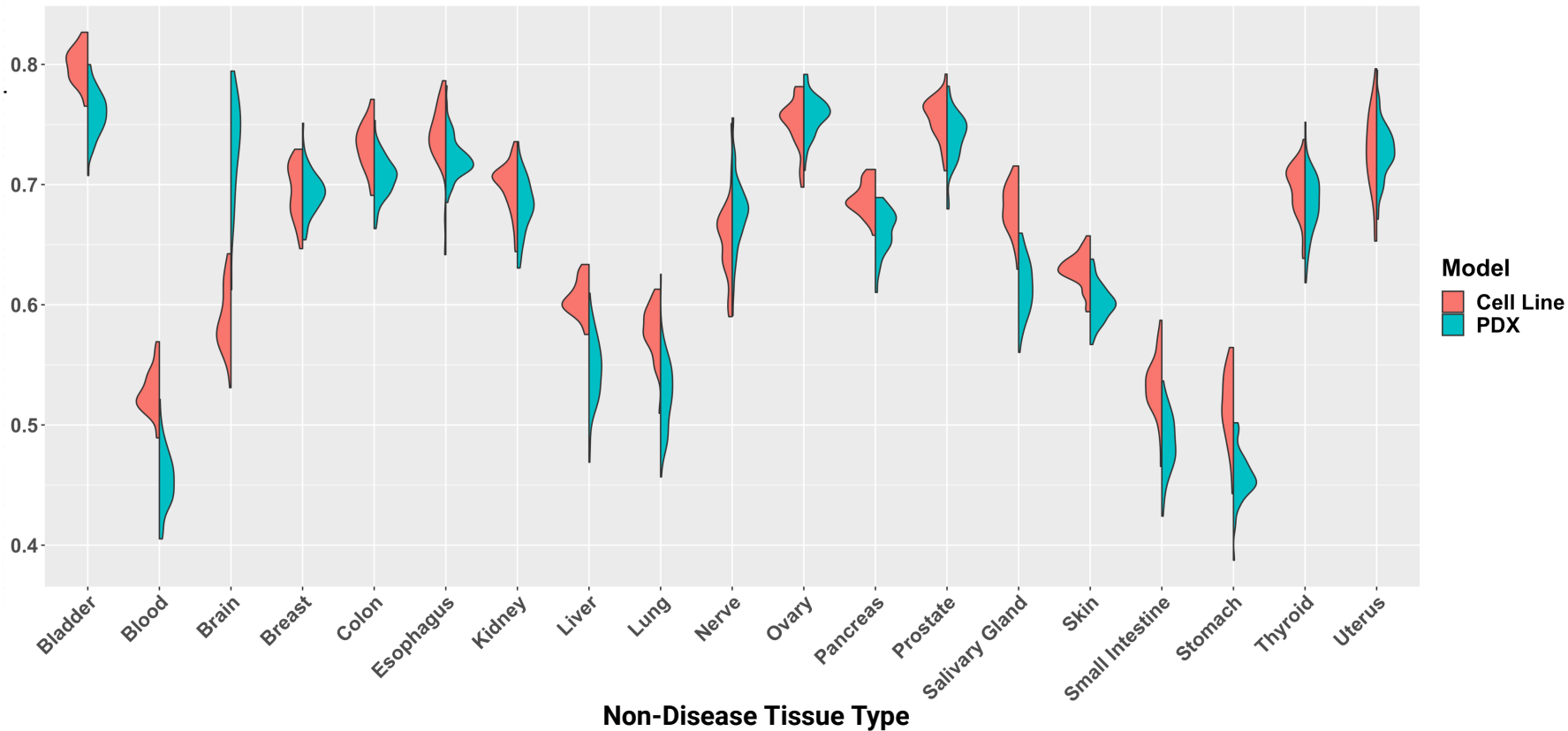

Supplement: Supplementary file 3 — FIGURE S3. Histogram of the distribution of Spearman's correlation rho between A. GBM cell lines and brain tumor tissue, B. GBM cell lines and brain non‐diseased tissue, C. GBM PDXs and brain non‐diseased tissue, and D. GBM PDXs and brain tumor tissue, respectively. [file CNR2-6-e1874-s007.pdf]

A

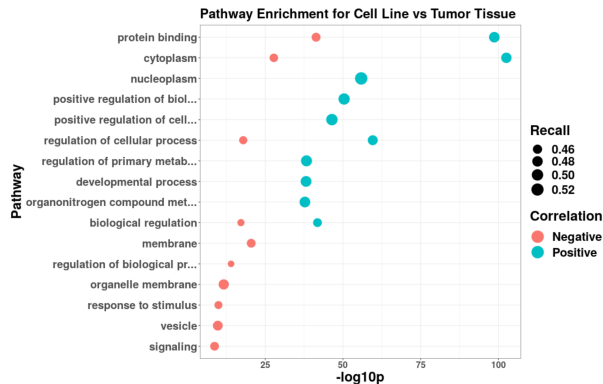

B

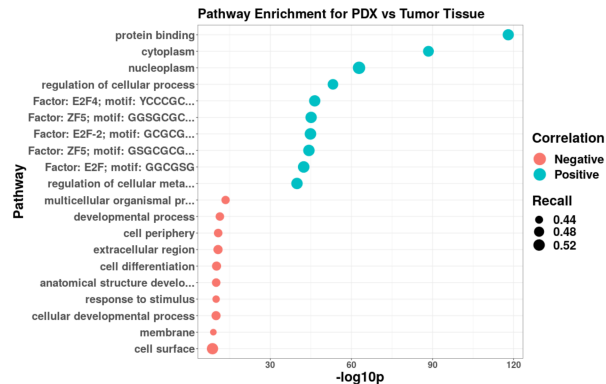

C

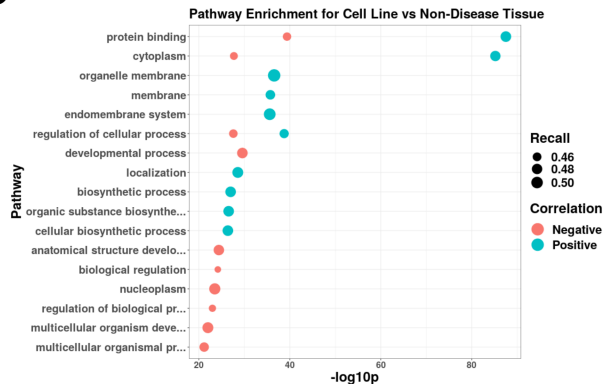

D

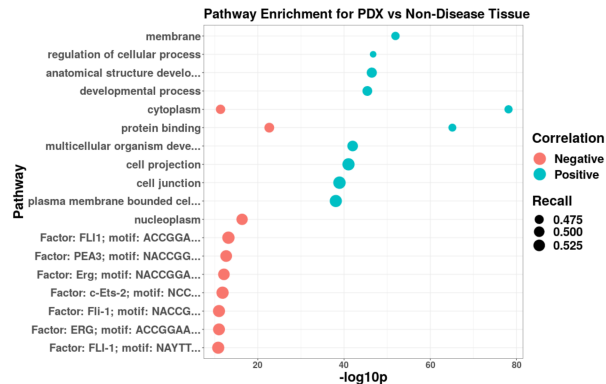

Supplement: Supplementary file 4 — FIGURE S4. Dot plots of the most significant (top 10 if at least 10 were significant) pathways represented in insignificant genes with either positive (blue) or negative (red) correlation between A. GBM cell lines and brain tumor tissue, B. GBM PDXs and brain tumor tissue, C. GBM PDXs and brain non‐diseased tissue, and D. GBM cell lines and brain non‐diseased tissue, respectively. Dots are sized by recall or the proportion of genes from a specific term represented in the data divided by the total genes possible for that term. [file CNR2-6-e1874-s002.pdf]
