# Supplementary material for: Evaluating cancer cell line and patient‐derived xenograft recapitulation of tumor and non‐diseased tissue gene expression profiles in silico
Source: Cancer Rep (Hoboken). 2023 Aug 2;6(9):e1874. doi: 10.1002/cnr2.1874 (PMC10480419; doi:10.1002/cnr2.1874)
Supplement: Supplementary file 2 — FIGURE S2. Split violin plots of the correlation of each GBM cancer cell line and PDX sample to each non‐diseased tissue type by that tissue's tissue‐specific genes. [file CNR2-6-e1874-s006.pdf]

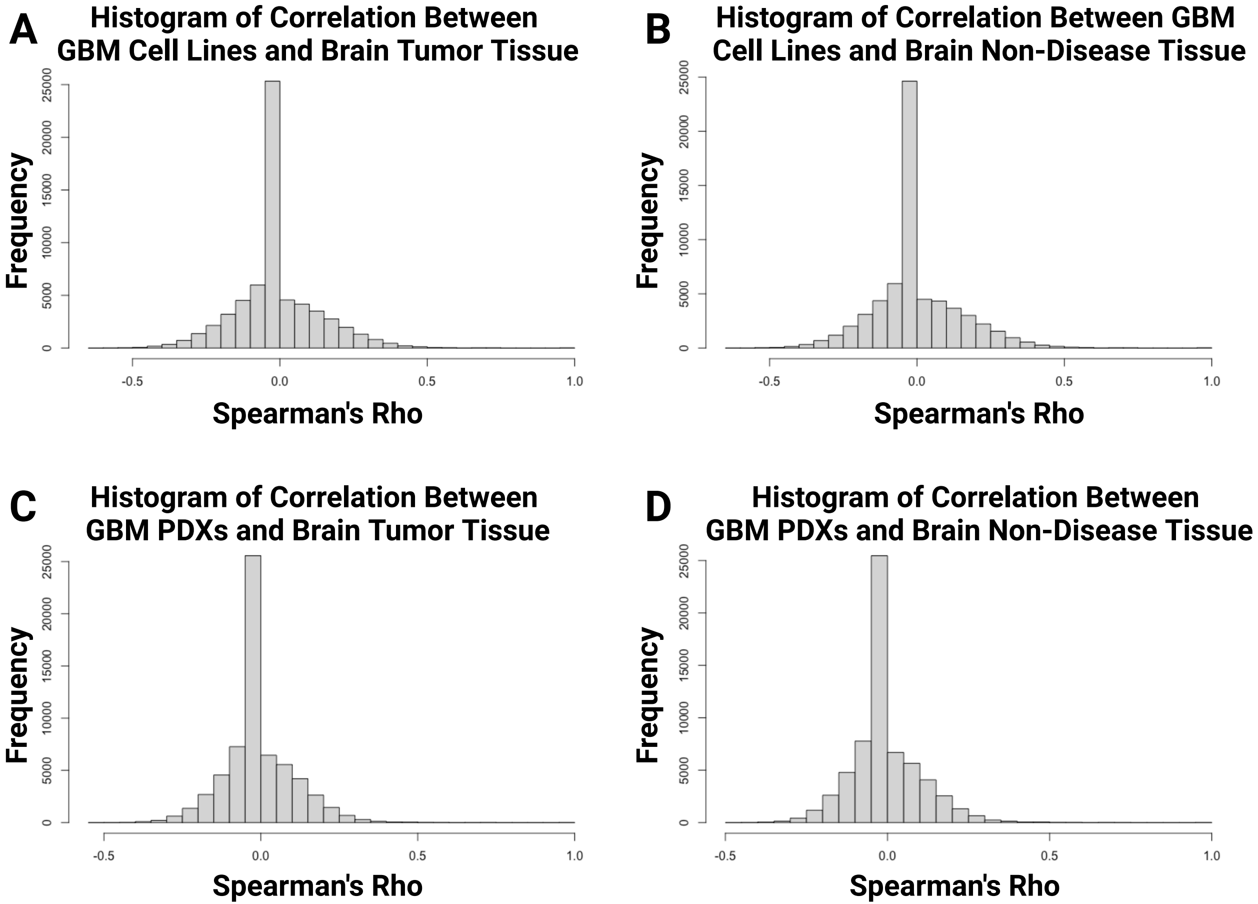

A
Histogram of Correlation Between
GBM Cell Lines and Brain Tumor Tissue
Frequency
0
5000
10000
15000
20000
25000
-0.5
0.0
0.5
1.0
Spearman's Rho
B
Histogram of Correlation Between GBM
Cell Lines and Brain Non-Disease Tissue
Frequency
0
5000
10000
15000
20000
25000
-0.5
0.0
0.5
1.0
Spearman's Rho
C
Histogram of Correlation Between
GBM PDXs and Brain Tumor Tissue
Frequency
0
5000
10000
15000
20000
25000
-0.5
0.0
0.5
1.0
Spearman's Rho
D
Histogram of Correlation Between
GBM PDXs and Brain Non-Disease Tissue
Frequency
0
5000
10000
15000
20000
25000
-0.5
0.0
0.5
1.0
Spearman's Rho
